# High Sinus, Lower Risk: A Paradigm Shift in Frontal Fracture Patterns

**DOI:** 10.1111/coa.70157

**Published:** 2026-08-13

**Authors:** Shmuel Wechsler, Haim Gavriel, Limor Muallem Kalmovich, Itzhak Aronov, Uri Chavkin

**Affiliations:** ^1^ Department of Otolaryngology–Head and Neck Surgery Shamir (Assaf Harofeh) Medical Center Zeriffin Israel; ^2^ Gray Faculty of Medical and Health Sciences Tel‐Aviv University Tel‐Aviv Israel

## Abstract

**Objective:**

The morphology of the frontal sinus (FS) has long been debated as a determinant of fracture type and severity. This study aimed to evaluate the association between sinus dimensions, fracture patterns, and clinical outcomes using real‐world data.

**Study Design:**

Retrospective cohort study.

**Setting:**

Tertiary trauma referral center.

**Methods:**

We retrospectively reviewed the records of 48 frontal sinus fractures treated at a tertiary trauma center. Volumetric and dimensional measurements (volume, height, width, and anteroposterior depth) were obtained from CT scans. Each sinus was segmented and analyzed separately using 3D Slicer software. Sinuses were categorised according to cohort‐derived percentiles and established classifications. Associations between sinus anatomy, fracture type, and outcomes were analyzed using logistic regression.

**Results:**

While most sinus dimensions showed odds ratios greater than 1 for anterior table fractures, only the categorical analysis of sinus height demonstrated statistical significance. Cohort‐based high sinuses (≥ 22.22 mm) were associated with isolated anterior table fractures (*p* = 0.014, OR = 4.83, 95% CI: 1.38–16.9). When comparing sinuses with larger volumes, a similar trend towards anterior‐only fractures was observed, though not statistically significant. Patients with anterior‐only fractures had fewer displaced fractures, shorter hospitalisation, and lower rates of intracranial haemorrhage and ICU admission.

**Conclusion:**

Frontal sinus height appears to be the most clinically relevant parameter influencing fracture patterns. Vertically expanded sinuses may act as protective “crumple zones,” mitigating posterior table involvement and severe intracranial injury.

## Key Points

- Frontal sinus height ≥ 22.22 mm is significantly associated with isolated anterior table fractures (OR = 4.83, *p* = 0.014), while overall sinus volume shows no statistically significant association with fracture type.
- Patients with isolated anterior table fractures had markedly better clinical outcomes, including shorter median hospitalisation (5 vs. 10 days), lower ICU admission rates (18% vs. > 50%), and less intracranial haemorrhage compared with those with posterior table involvement.
- Vertically expanded frontal sinuses may function as protective “crumple zones” by deflecting anterior–posterior impact forces superiorly, thereby reducing stress transmitted to the posterior table and intracranial structures.
- Neither sinus width nor depth was significantly associated with fracture type in this cohort, identifying height as the dominant protective dimensional parameter of frontal sinus morphology.
- These findings may inform risk stratification for individuals at elevated risk of head trauma (e.g., contact‐sport athletes, boxers, professional drivers) and guide the design of protective headgear targeting craniofacial impact mitigation.

## 1 Introduction

The frontal sinuses (FS) are absent at birth and begin to form only at the age of two years [1]. Their full development occurs at the ages of 14 years, the last of the paranasal sinuses to complete development [2]. About 5%–15% of the population does not have FS, and 4%–5% have only small rudimentary air cells [3, 4].

Each FS is shaped like a pyramid, with its base directed medially to form the intersinus septum. Its “Outer” or Anterior Table (AT) is strong and sturdy, with an average thickness of 4 mm to 12 mm [5].

The force required to fracture the frontal bone is between 800 and 1600 pounds [6]. Fractures of the frontal bone with associated involvement of the FS account for approximately 5%–8% of all maxillofacial injuries [7].

Fractures of the FS can be broadly divided into Isolated AT Fracture (ATF) [8], combined anterior and posterior table (PT) fractures and isolated PT fractures. The fracture pattern and its complexity depend on many factors, such as the impact force intensity and direction, site of impact, and frontal bone quality. However, the influence of frontal sinus volume (FSV) on stress distribution and fracture patterns during impact within the frontal region remains unclear [9].

The volume of the FS varies widely among individuals, and several classification systems have been proposed to categorise sinus aeration. Guerram et al. classified FS into four categories: (1) aplasia, (2) hypoplasia, sinus limited to the area below the supraorbital line; (3) medium size, sinus extending medially to the midorbital line; and (4) hyperplasia, sinus extending laterally beyond the midorbital line [10]. Aslier et al. proposed a volume‐based classification on cadavers: hypoplastic (< 1131.25 mm^3^), normal (1131.25–3328.50 mm^3^), and hyperplastic (> 3328.50 mm^3^). Cohen et al. measured 2.92 mL as the mean volume of FS in their cohort [11], and Dhakal et al. showed in their cohort that normal range for sinus is 2.59 mL with IQR of 1.42–4.59 [12].

The potential influence of FSV on fracture patterns after frontal impact has been the subject of biomechanical research. Kellman and Schmidt hypothesised that the paranasal sinuses act as “crumple zones,” serving as compressible structures that absorb and dissipate impact energy through deformation or fracture of their bony walls [13].

This study aimed to evaluate correlation between FSV or FS dimensions and fracture types in a real‐world clinical setting.

## 2 Materials and Methods

This study was approved by the Institutional Review Board (Helsinki Committee) of Shamir (Assaf Harofeh) Medical Center under approval number 0091‐23ASF (14/06/2023). This study included all patients diagnosed with FS fractures at a tertiary Medical Center between 2010 and 2024. Patients were identified through a review of electronic medical records and radiology databases. The inclusion criteria were age ≥ 14 years, confirmed FS fracture on computed tomography (CT), and complete medical and radiological data. Patients with prior FS surgery, previous fractures, or incomplete medical records were excluded from the study. The data collected included demographic information, mechanism of injury, anatomical characteristics of the FS, including its dimensions and volume, associated cranial fractures, intracranial injuries, hospital course including intensive care unit (ICU) admission, and surgical intervention.

Mechanisms of injury were categorised based on the anticipated energy of the impact. Low‐energy trauma, such as simple ground‐level falls (e.g., tripping) and physical altercations, was classified as simple blunt trauma. High‐energy trauma was subdivided into motor vehicle collisions and falls from a significant height (defined as a fall from above the first floor).

The CT imaging protocols for head injury in the ER unit were as follows: 120 kVp, 256 mAs; slice thickness, 1 mm; slice range, 1 mm; FOV, 24 cm; and matrix, 512 × 512. From axial planar images obtained and transferred from ‘PACS’ (Picture Archiving and Communication System) software to the publicly available Slicer 3D software (version 5.6.3) using ‘DICOM’ (Digital Imaging and Communications in Medicine) format for segmentation of bony compartments and definition of the regions of interest. Sagittal and coronal reformatted images were created. The area of interest was determined by manual tracing using a mouse‐guided cursor.

We measured each sinus separately using three‐dimensional volumetric analysis. To ensure reliability, the manual segmentation of the frontal sinus boundaries, from the frontal beak to the sinus roof and lateral wall, and the subsequent volumetric measurements were performed concurrently by two otolaryngologists. It was established a priori that any discrepancy in volume measurement greater than 0.5 mL between the two reviewers would be resolved by consulting a third senior expert. After extraction of the FSV, the maximum dimensions of each axis were measured, including the FS height (FSH)—measured from sinus roof to sinus floor, FS width (FSW)—measured from sinus septum to sinus lateral wall, and FS depth (FSD)—measured from AT to PT. If ethmoidal cells were present within the FS, they were included in the total FSV measurement (Figure 1).

**FIGURE 1:**
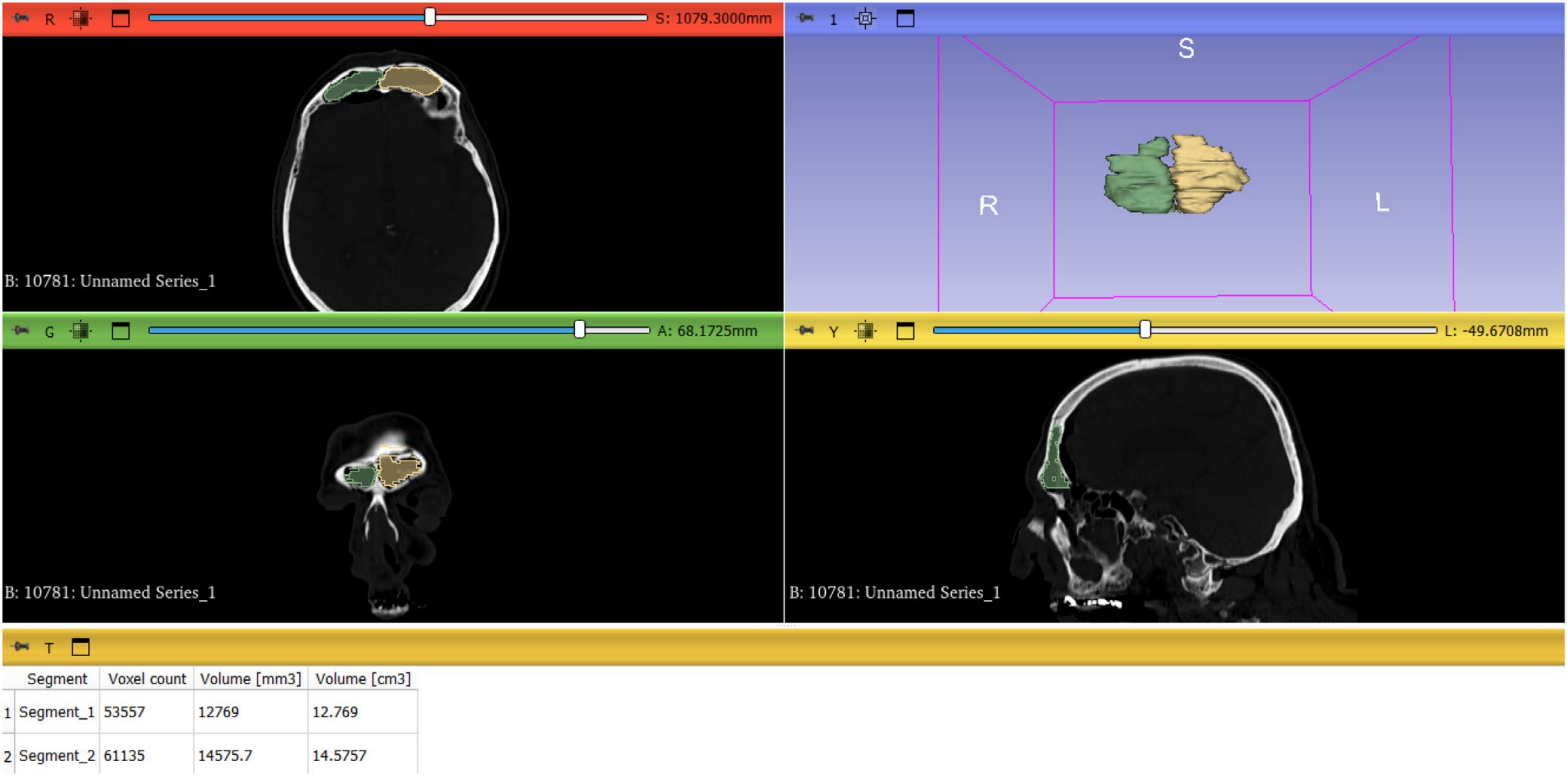
Measurement of frontal sinus volume using 3D Slicer software. Representative CT image analyzed in 3D Slicer (version 5.6.3) showing the segmentation of frontal sinuses. The right (green) and left (yellow) sinuses were outlined manually in the axial, coronal, and sagittal planes with a corresponding 3D reconstruction. Volumes were calculated automatically from the voxel counts and expressed in millilitres (mL).

### 2.1 Statistical Analysis

Data were analysed using JASP (version 0.95; JASP Team, Amsterdam, Netherlands). The distribution of continuous variables was assessed using the Shapiro–Wilk test. Normally distributed data are presented as mean ± standard deviation (SD), whereas skewed data are summarised as median with interquartile range (IQR). Categorical variables are reported as absolute counts and percentages.

To explore the independent predictors of fracture type, separate binary logistic regression models were constructed for anterior, combined, and PT fractures and isolated ATF versus PT involvement. Odds ratios (OR) with corresponding 95% confidence intervals (CI) were calculated. Statistical significance was set at *p* < 0.05.

For the analysis of sinus anatomy, FSV was categorised according to the classification by Aslier et al. hypoplastic (< 1131.25 mm^3^), normal (1131.25–3328.50 mm^3^), and hyperplastic (> 3328.50 mm^3^) [14], and Dhakal et al. classification (normal = 1.42–4.59) and another categorisation according to the study cohort tertiles and later according to volume quartiles. These categorical variables were incorporated into the logistic regression models to assess their association with fracture type.

## 3 Results

A total of 48 FS fractures were eventually included from 39 patients. Nine patients (23%) sustained bilateral fractures, while the remaining 30 had unilateral involvement. The median patient age was 34 years (IQR 20–74), with those sustaining isolated ATF being generally older than patients with PT involvement. The cohort was predominantly male (84.6%). The most common mechanism of injury was simple blunt trauma (53.8%), representing low‐energy impacts such as ground‐level falls and altercations, followed by high‐energy impacts including falls from a significant height (27.5%) and motor vehicle collisions (23.1%).

Intracranial haemorrhage (ICH) was identified in 47.1% of all fractures and was least common in ATF (31.8%) compared with combined (63.6%) and PF (42.9%) (OR = 0.33, *p* = 0.061). (Table 1).

**TABLE 1:** Characteristics of patients with frontal sinus fractures.

Characteristic	Total (*n* = 39)	
Age, years median (IQR)	34 (20–74)	
Gender	Male	33 (84.6%)
	Female	6 (15.4%)
Trauma mechanism	Motor Vehicle Accidents	9 (23.1%)
	Falls From Height	9 (27.5%)
	Simple Blunt Trauma	21 (53.8%)
Anticoagulant	6 (15.4%)	
Fracture side	Right	18 (46%)
	Left	12 (31%)
	Bilateral	9 (23%)
Intracranial haemorrhage	24 (47.1%)	
Total associated fractures median (IQR)	3 ± 2.25	
Admission	37 (95%)	
Median days of hospitalisation (IQR)	4 (1.5–8)	
ICU admission	14 (36%)	
Median days of ICU (IQR)	5 (2–16.5)	

Abbreviation: ICU, intensive care unit.

Only 18% of patients with isolated ATF required ICU admission, in contrast to more than half of those with combined or posterior involvement (OR = 0.12, *p* = 0.003). Hospitalisation duration was also shorter for the anterior‐only group (median 5 days) compared with patients with posterior or combined fractures (median 10 days; OR = 0.90, *p* = 0.036).

When analysing the relationship between sinus dimensions and fracture type, no significant correlation was found between PT involvement and overall FSV (OR = 1.06, *p* = 0.787), depth (OR = 1.06, *p* = 0.676), or height as a continuous variable (OR = 1.08, *p* = 0.157) (Table 2, Figures 1 and 2).

**TABLE 2:** Frontal sinus dimensions and classifications.

Variable	Total (*n* = 48)	Isolated anterior table (*n* = 21)	Posterior table involvement (*n* = 27)	*p*	OR (anterior vs. others)	95% CI
Continuous measures						
Volume (mL)	4.80 ± 3.13	5.6 (2.1–7.5)	3.7 (2.2–6.0)	0.787	1.06	(0.68–1.64)
Height (mm)	25.17 (18.6–30.3)	30.04 (19.1–37.6)	23.2 (18.4–28.9)	0.157	1.08	(0.97–1.21)
Width (mm)	33.3 (25.5–40.6)	34.3 (27.4–40.7)	32.4 (25–40.7)	0.335	0.95	(0.84–1.08)
Depth (mm)	10.8 (8.0–14.2)	12.39 (8.5–15.3)	10.06 (8–13)	0.676	1.06	(0.82–1.38)
Categorical measures						
Dhakal—hyperplastic (> 4.59 mL)		13 (62%)	10 (32%)	0.087	2.76	(0.86–8.87)
Dhakal—normal + hypoplastic		8 (38%)	17 (68%)			
Aslier—hyperplastic (> 3328.50 mm^3^)		13 (46%)	15 (40%)	0.658	1.27	(0.43–3.73)
Aslier—normal + hypoplastic		8 (54%)	12 (60%)			
Cohort depth—high (> 12.69 mm)		9 (43%)	7 (26%)	0.217	2.14	(0.63–7.23)
Cohort depth—normal + low		12 (57%)	20 (74%)			
Cohort width—high (> 37.91 mm)		8 (38%)	8 (30%)	0.537	1.40	(0.42–4.70)
Cohort width—normal + low		13 (62%)	19 (70%)			
Cohort height—high (≥ 22.22 mm)		11 (52%)	5 (19%)	0.014*	4.83	(1.38–16.9)
Cohort height—normal + low		10 (48%)	22 (81%)			
Cohort volume Q4—hyperplastic (> 6.7 mL)		8 (66%)	4 (33%)	0.065	3.54	(0.79–15.8)
Cohort volume Q4—normal + hypoplastic		13 (36%)	23 (64%)			

*Note:* Odds ratios (OR) were calculated for anterior table–only fractures versus all other fracture types. Categorical groupings of sinus dimensions were cohort‐based.

* *p* < 0.05.

**FIGURE 2:**
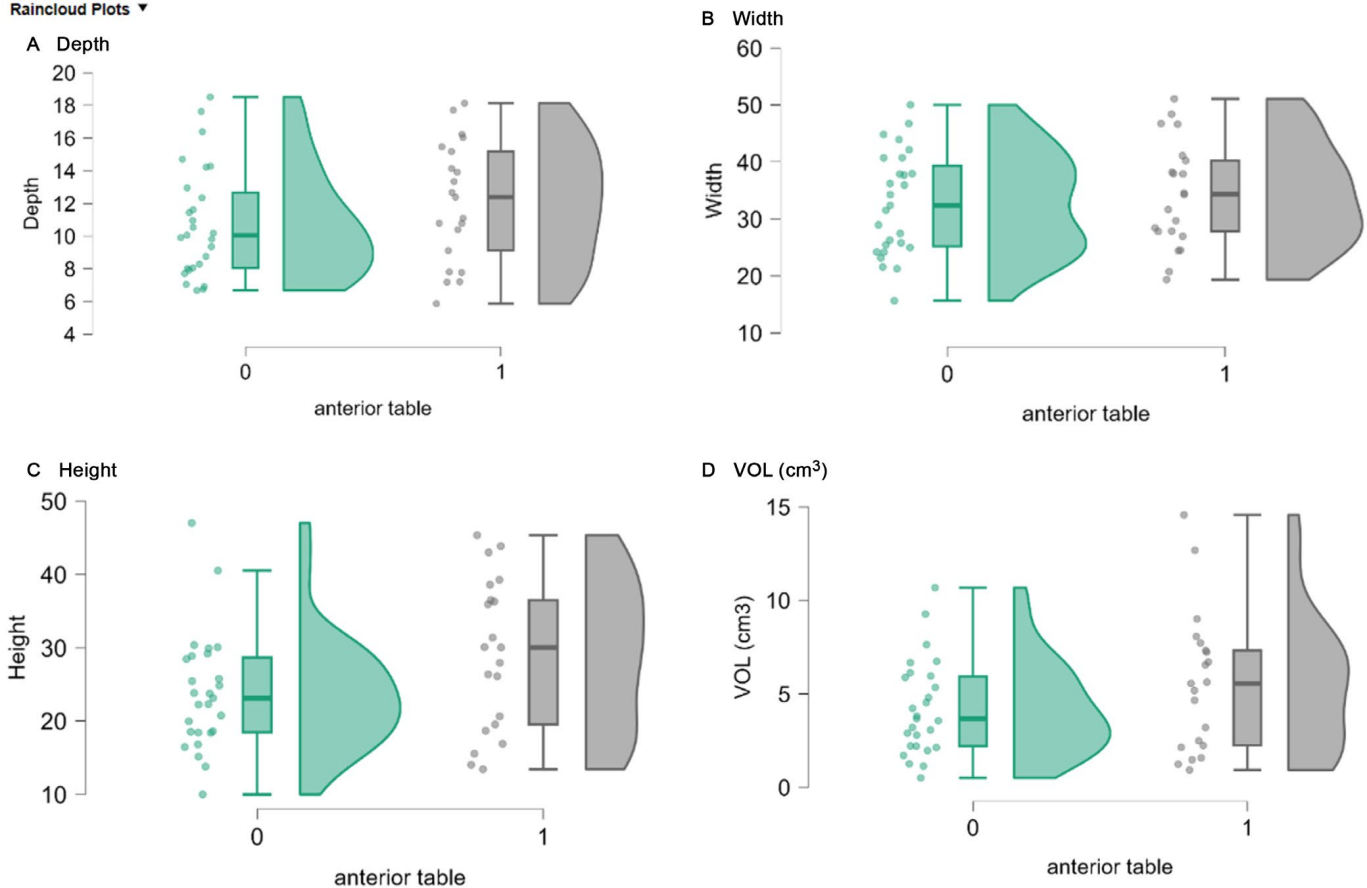
Distribution of Frontal Sinus (FS) Dimensions by Presence of Anterior Table Fracture. Violin plots with overlaid boxplots and individual data points illustrating the distribution of frontal sinus measurements according to the presence (1) or absence (0) of only anterior table involvement. (A) frontal sinus depth dimension (mm). (B) Sinus width dimension (mm). (C) Sinus height (mm). (D) Sinus volume (VOL, mL). Each plot shows the median, interquartile range, and kernel density estimation of the distributions. For all measured dimensions, the odds ratios (OR) for anterior table fractures were greater than 1 but *p*‐value > 0.05.

We examined FSV using volumetric classifications as well as cohort‐derived cutoffs. Across all tested thresholds, including Aslier's (> 3328.50 mm^3^) and Dhakal's hyperplasia criterion (> 4.59 mL), sinus volume did not demonstrate a statistically significant association with fracture type. At the upper extreme of sinus volume, a consistent trend was noted, with markedly enlarged sinuses showing higher odds of isolated ATF at higher cutoffs (OR≈3.54 for volumes > 6.7 mL; *p* = 0.065).

We next assessed individual sinus dimensions. Categorical analysis of sinus height showed a significant association: high sinuses (≥ 22.22 mm) were more likely to sustain isolated ATF compared with normal or low sinuses (52% vs. 19%; OR = 4.83, 95% CI: 1.38–16.9; *p* = 0.014) (Figure 3). Representative examples of isolated anterior table, combined anterior–posterior table, and isolated posterior table fractures are shown in Figure 4.

**FIGURE 3:**
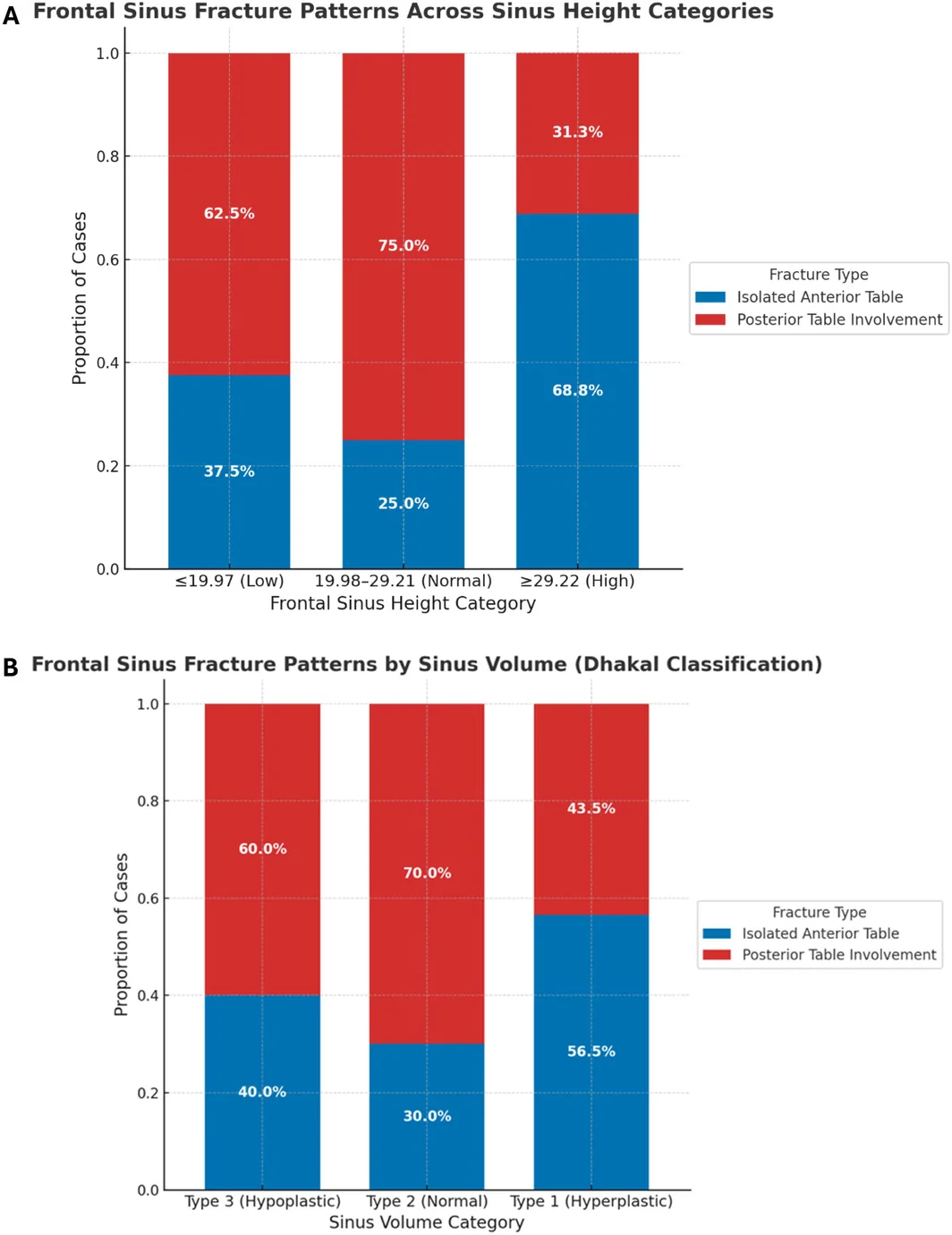
Stacked bar charts showing the proportions of fracture patterns across frontal sinus anatomical dimensions. (A) Cohort‐based high sinuses were most frequently associated with isolated anterior table fractures (*p* = 0.014), whereas posterior table involvement was predominantly observed in sinuses of normal or low height. (B) Hyperplastic sinuses (Dhakal classification) showed a trend towards a higher frequency of anterior table fractures compared with normal and hypoplastic sinuses, which more often demonstrated posterior table involvement (*p* = 0.087).

**FIGURE 4:**
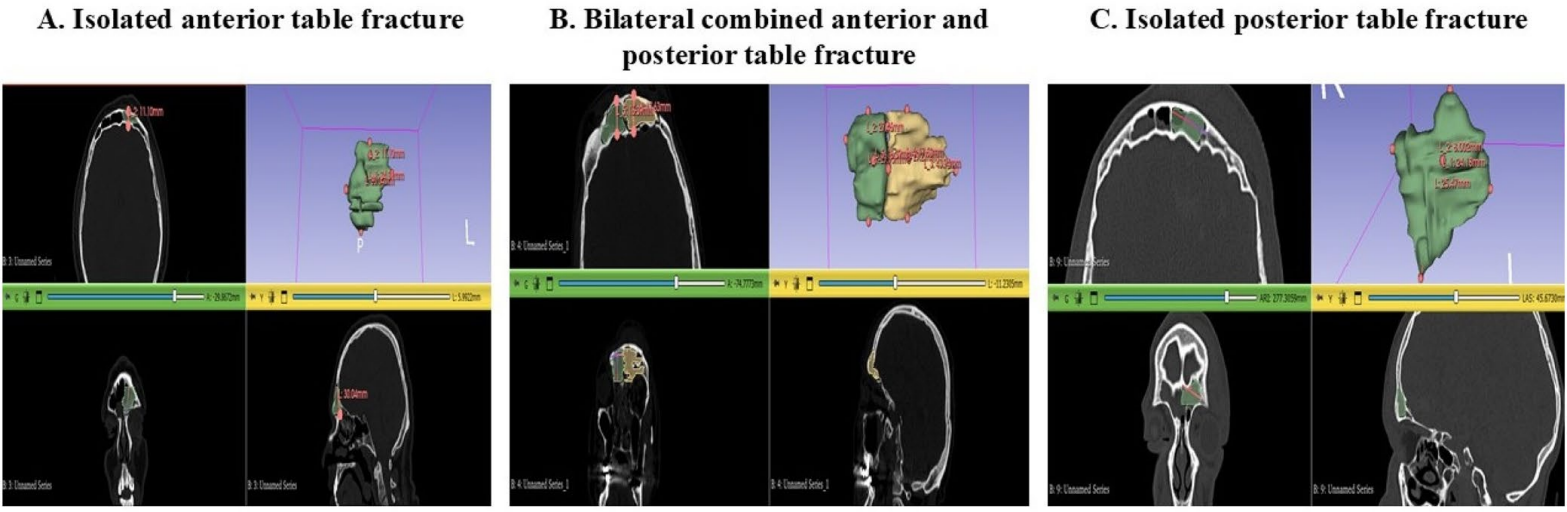
Representative frontal sinus fracture patterns. (A) Isolated anterior table fracture (frontal sinus height 30.0 mm, volume 5.6 cm^3^). (B) Bilateral combined anterior and posterior table fracture (maximum frontal sinus height 27.5 mm, volume 7.6 and 9.3 cm^3^). (C) Isolated posterior table fracture (frontal sinus height 8.0 mm, volume 2.2 cm^3^). Original CT images and three‐dimensional reconstructions generated using 3D Slicer are shown without modification.

## 4 Discussion

ATF are generally less severe injuries, resulting primarily in aesthetic deformities. In contrast, PT‐involving fractures are more prone to serious complications such as pneumocephalus, cerebrospinal fluid (CSF) leakage, and epidural hematoma [15]. In our cohort, patients with isolated ATF tended to be older and demonstrated a milder clinical course, characterised by lower rates of intracranial haemorrhage, reduced ICU admission, and shorter hospital stays. Conversely, combined and PT fractures were associated with higher ICU admission rates, intracranial haemorrhage, and prolonged hospitalisation.

Several authors have examined the relationship between FS size and fracture pattern. Buller et al. reported associations between fracture type and sinus width, height, and depth, measured by combining both sinuses into a single unit [16]. They observed that larger sinuses were more likely to sustain isolated ATF, whereas hypoplastic and medium‐sized sinuses were more often involved in combined or PT fractures. In a separate study, Buller et al. found that sinus hyperplasia was associated with a markedly increased risk of ATF [17]. Notably, both studies relied on single‐dimensional or qualitative classifications and did not incorporate true three‐dimensional volumetric analysis. Our study quantitatively evaluated the relationship between FSV, its dimensions, and fracture patterns using three‐dimensional segmentation.

Our analyses showed that FSV did not demonstrate a statistically significant association with fracture patterns. Nonetheless, at the upper extreme of sinus size, we observed a trend towards isolated ATF. This stepwise behaviour suggests that only markedly enlarged sinuses may meaningfully influence fracture distribution.

In contrast, sinus height showed a clear and statistically significant association with fracture type. High sinuses (≥ 22.22 mm) were significantly associated with isolated ATF in 68.8% (*p* = 0.014, OR = 4.83; Figure 3). These findings suggest that the vertical dimension plays a dominant role in dissipating traumatic energy and protecting posterior structures. In contrast to sinus height, neither sinus depth nor width showed any significant effect on fracture type.

Several biomechanical and clinical studies have addressed the potential protective role of FS morphology [18]. Pajić et al., using finite element analysis, demonstrated that well‐developed sinuses function as shock absorbers, protecting the brain and posterior wall by fracturing anteriorly. Similarly, Yu et al. reported that patients with larger FS had a 33% lower risk of brain contusion compared with those with smaller sinuses [19].

Our findings refine the “crumple zone” theory proposed by Kellman and Schmidt, by highlighting that the vertical dimension of the FS, rather than its overall volume, is the principal determinant of this protective capacity.

When considering why height, but not width or depth, was associated with this protective effect, we speculated that the advantage of a tall sinus may not stem solely from a greater area for energy absorption. Rather, in vertically elongated sinuses, the PT forms a steeper angle relative to the typical anterior–posterior impact vector, allowing a portion of the force to be deflected superiorly instead of directly backward [20].

These insights may be particularly relevant for individuals at increased risk of head trauma, such as athletes in contact sports, boxers, and professional drivers. Identifying patients with low or normal FS development may help stratify their susceptibility to PT fractures or brain contusion, while recognising those with tall or hyperplastic sinuses as relatively protected. A better understanding of sinus morphology could therefore inform radiologic evaluation, surgical planning, and the design of protective strategies or headgear aimed at mitigating craniofacial impact.

This study's limitations include a retrospective, single‐center design and a small sample size that restricted multivariable modelling. Crucially, we could not precisely quantify the impact force. While we stratified injuries into low‐energy (e.g., ground‐level falls, assaults) and high‐energy (e.g., falls > 1st floor) mechanisms to partially address this, isolated anterior table fractures might still reflect lower‐energy trauma rather than solely the sinus's protective “crumple zone” effect. Furthermore, manual segmentation variability and a lack of correlation adjustments for bilateral cases remain minor limitations. Given the heterogeneity inherent to facial trauma, larger multicenter biomechanical studies are needed to generalise and fully validate these findings.

## Author Contributions

**Shmuel Wechsler:** conceptualization, methodology, investigation, data curation, formal analysis, statistical analysis, visualization, writing – original draft, writing – review and editing. **Uri Chavkin:** conceptualization, supervision, project administration, writing – review and editing. **Itzhak Aronov:** data curation, imaging analysis, three‐dimensional volumetric measurements, writing – review and editing. **Limor Muallem Kalmovich:** writing – review and editing. **Haim Gavriel:** conceptualization, supervision, writing – review and editing. All authors reviewed and approved the final version of the manuscript.

## Funding

The authors have nothing to report.

## Ethics Statement

This study was approved by the Institutional Review Board (Helsinki Committee) of Shamir Medical Center under approval number 0091‐23ASF (14/06/2023).

## Conflicts of Interest

The authors declare no conflicts of interest.

## Data Availability

The data that support the findings of this study are available from the corresponding author upon reasonable request. The data are not publicly available because they contain information that could compromise participant privacy or are subject to institutional ethical restrictions.
